# Virtual Screening, Molecular Docking and Molecular Dynamics Simulation of Bioactive Compounds from Various Indonesian Medicinal Plants as Potential Inhibitors of Human Papillomavirus Type 16 E6 Protein in Cervical Cancer Development

**DOI:** 10.21315/tlsr2025.36.1.1

**Published:** 2025-03-30

**Authors:** Arief Hidayatullah, Diana Widiastuti, Wira Eka Putra, Muhaimin Rifa’i, Muhammad Fikri Heikal, Sustiprijatno

**Affiliations:** 1 Democratic Governance and Poverty Reduction Unit, United Nations Development Programme, Eijkman-RSCM Building, Jl. Diponegoro 69, 10430 Jakarta, Indonesia; 2 Department of Chemistry, Faculty of Mathematics and Natural Sciences, Universitas Pakuan, Jl. Pakuan, RT.02/RW.06, 16129 Tegallega, Bogor, West Java, Indonesia; 3 Biotechnology Study Program, Department of Applied Sciences, Faculty of Mathematics and Natural Sciences, Universitas Negeri Malang, Jl. Semarang 5, 65145, Malang, East Java, Indonesia; 4 Department of Biology, Faculty of Mathematics and Natural Sciences, Brawijaya University, Jl. Veteran, 65145 Malang, East Java, Indonesia; 5 Department of Biology, Faculty of Mathematics and Natural Sciences, Universitas Negeri Malang, Jl. Semarang 5, 65145 Malang, East Java, Indonesia; 6 Research Center for Applied Botany, National Research and Innovation Agency, Jl. Raya Jakarta-Bogor Km 46, 16911 Cibinong-Bogor, West Java, Indonesia

**Keywords:** Cervical Cancer, Dynamics Simulation, E6 Protein, HPV16, Molecular Docking

## Abstract

Infection of keratinocytes by high-risk human papillomavirus (HPV) strains, notably HPV16, is responsible for the onset of cervical cancer. The E6 protein serves as a pivotal oncoprotein implicated in the progression of cancer. We utilised a virtual screening method to identify bioactive compounds in a variety of commonly used medicinal plants in Indonesia. All the top five compounds bind to a single binding site on the E6 major hydrophobic groove, which corresponds to the binding site for the E6AP and IRF3’s LxxLL motifs. They are expected to function as competitive inhibitors, inhibiting the development of the E6-E6AP and E6-IRF3 complexes, which limit p53 degradation and therefore cell proliferation, thus preserving the innate immune response to HPV16 infection. Asarinin and thiazolo[3,2-a]benzimidazole-3(2H)-one,2-(2-fluorobenzylideno)-7,8-dimethyl were predicted to be the most effective compounds in this research owing to their strong affinity for and persistent interactions with the E6 major hydrophobic groove, particularly in comparison to pharmacological controls.

**Highlights**

- The E6 protein serves as a pivotal oncoprotein implicated in cancer progression.
- Asarinin and thiazolo[3,2-a]benzimidazole-3(2H)-one,2-(2-fluorobenzylideno)-7,8-dimethyl demonstrated strong affinity and stable interaction against HPV16 E6 protein.
- Asarinin and thiazolo[3,2-a]benzimidazole-3(2H)-one,2-(2-fluorobenzylideno)-7,8-dimethyl were predicted to be the most effective compounds for HPV16 E6 protein inhibitor candidate.

## INTRODUCTION

Human papillomavirus, or HPV, constitutes a cluster of non-enveloped DNA viruses that specifically assemble within the nucleus and predominantly target the basal layer of keratinocytes. Its transmission primarily occurs through skin-to-skin contact, notably via sexual contact (Bonnez 2009; Forcier & Musacchio 2010; Tao *et al*. 2003). Presently, there exist over 200 identified types of HPV, categorised generally into five genotype groups (α, β, γ, μ and ν) and classified into two risk categories: high-risk (HR) and low-risk (LR) HPV (Bzhalava *et al*. 2013; Cobo 2012; Graham 2017). LR-HPV infections, responsible for the majority of HPV cases, are generally asymptomatic and pose no significant health risks. The primary manifestation of these infections is the development of skin warts on various body parts including the hands, feet and genital area. Conversely, HR-HPV infections, stemming from HPV types 16, 18, 31, 35, 39, 45, 51, 56, 59, 68, 73 and 82, serve as the principal precursors to anogenital cancers, with cervical cancer being the most prevalent among them (Bzhalava *et al*. 2013; Doorbar *et al*. 2012; Graham 2017; Tyring *et al*. 2016). Cervical cancer stands as one of the most prevalent malignancies affecting women globally. In Indonesia specifically, cervical cancer ranks as the second most common cancer, following breast cancer, boasting a prevalence rate ranging between 43.3 and 52.4 cases per 100,000 individuals (Arbyn *et al*. 2020; Putri *et al*. 2019; Wahidin *et al*. 2020; Zhang *et al*. 2020). Around 70% to 90% of cervical cancer instances arise from infections attributed to HPV16 and HPV18, two prominent high-risk HPV strains. Notably, HPV16 alone is responsible for approximately 55% of all cervical cancer cases (Graham 2017; Huibregtse *et al*. 1993; Tan *et al*. 2012; Wang *et al*. 2018).

Regarding the extensively studied HPV type, HPV16, its viral genome comprises two distinct categories of genes. The early genes, which encompass the primary nonstructural proteins (E1, E2, E4, E5, E6 and E7), play pivotal roles in orchestrating the replication and maturation phases of HPV within host cells. Conversely, the late gene primarily oversees the assembly of mature virion structures, serving as the principal structural proteins (L1 and L2). Among those nonstructural proteins, one of the most studied is the E6 protein. The E6 protein is one of the critical oncoproteins that differentiate HR and LR variants of HPV (Doorbar *et al*. 2012; Egawa & Doorbar 2017; Graham 2017; Underbrink *et al*. 2016). In alpha-HR-HPV such as HPV16, this specific protein will inhibit the transactivation process and degradation of p53 using its 26S proteasome for degradation, causing DNA damage resulting in immortalisation of infected cells, leading to cancer development (Bernard *et al*. 2011; Doorbar *et al*. 2012; Scheurer *et al*. 2005). In addition, E6 is also known to inhibit innate immune responses to infected cells by inhibiting IRF3 and TYK2 signaling mechanisms (Gutiérrez-Hoya & Soto-Cruz 2020; Reiser *et al*. 2011; Tummers & van der Burg 2015). In its mechanism of action as an oncoprotein, E6 forms a dimer with E6-associated protein (E6AP) or IRF3 (Doorbar *et al*. 2012; M. Shah *et al*. 2013; Tummers & van der Burg 2015; Zanier *et al*. 2014). Interaction between E6 protein and E6AP is very crucial in the mechanism of cell immortalisation and cancer development; meanwhile, the E6 protein also binds to IRF3 and prevents its transcriptional activity, mainly the IFN-β mRNA synthesis, crucial to silence the innate immune responses (Ronco *et al*. 1998; Tummers & van der Burg 2015). We aimed to avoid this kind of formation through intervention by antiviral compounds that are competitive against E6AP and IRF3 (Reiser *et al*. 2011; Tan *et al*. 2012; Tummers & van der Burg 2015). Several anticancer drugs have E6 suppressant effects based on *in vitro* studies, including vorinostat and mitomycin C. Vorinostat functions as a competitive inhibitor at the E6 binding site of p53, while also downregulating the E6 protein itself in HPV-positive cervical cancer cells. As a pan-HDAC inhibitor, vorinostat exerts its effects by significantly reducing the activities of both E6 and E7 oncoproteins, crucial players in HPV-induced carcinogenesis. Additionally, vorinostat has been found to disrupt viral DNA amplification and inhibit host DNA replication, further impeding the progression of HPV-associated cervical cancer (Banerjee *et al*. 2018; Z. Lin *et al*. 2009; Tan *et al*. 2012). Mitomycin C exerts its effects as a DNA alkylating agent, suppressing E6 protein expression and disrupting its downstream effects. Specifically, mitomycin C is thought to inhibit the E6-activated RSV (Rous sarcoma virus) promoter. However, the precise mechanism by which mitomycin C achieves this inhibition is still not fully elucidated (Kang *et al*. 2010; Vande Pol & Klingelhutz 2013). A study mention that it could hinders the degradation process of p53, a tumour suppressor protein that is targeted for degradation by the E6 protein in HPV-infected cells (White *et al*. 2014).

Indonesia, being one of the largest tropical countries, hosts a rich diversity of over 7,000 species of medicinal plants. Despite this vast wealth, less than 10% of these species are officially acknowledged as phytopharmaceuticals (Salim & Munadi 2017). Throughout Indonesian culture, numerous medicinal plants have been utilised for centuries to manage or alleviate a spectrum of diseases, drawing upon empirical knowledge within communities. However, scientific substantiation for their efficacy sometimes remains limited (Jennifer & Saptutyningsih 2015; Sumayyah & Salsabila 2017). Around 55% of Indonesians use traditional medicines daily, and more than 95% of them thought they feel the benefits of these medicines (Jennifer & Saptutyningsih 2015; Sumayyah & Salsabila 2017). Those open up great opportunities to explore drug candidates for many diseases, including HR-HPV infection. Drug design research would be long-continuous, primarily if the ingredients used are natural-based. One of the earliest stages is the screening compound and trial based *in silico* as in this study (Putra *et al*. 2017; Kitchen *et al*. 2004; Lionta *et al*. 2014; Putra *et al*. 2020). Specifically, this study aims to explore potential anti-HPV E6 compounds derived from natural ingredients outside of the rhizome group because most research on herbal medicines and their uses has focused on them (Beers 2012; Hakim 2015; Kristianto *et al*. 2020; Woerdenbag & Kayser 2014). Preliminary studies showed 476 bioactive compounds from 18 non-rhizome simplicia that could be tested through virtual screening against HPV16 E6 protein (Abdallah *et al*. 2016; Abdel-Moneim *et al*. 2013; Amalina *et al*. 2013; Anwar *et al*. 2009; Asika *et al*. 2016; Badgujar *et al*. 2014; Beyzi *et al*. 2017; Choi *et al*. 2016; Chung 2009; Dertyasasa & Tunjung 2017; Ekpenyong *et al*. 2015; El-Saber Batiha *et al*. 2020; Fagodia *et al*. 2017; Figueirinha *et al*. 2008; González-Molina *et al*. 2010; Kadam & Lele 2017; Laribi *et al*. 2015; Li *et al*. 2017; Mahmood *et al*. 2016; Majdi *et al*. 2020; Mohammed *et al*. 2016; Pandey *et al*. 2016; Patil *et al*. 2009; Patra *et al*. 2020; Piras *et al*. 2013; Rattanachaikunsopon & Phumkhachorn 2010; G. Shah *et al*. 2011; Srivastava *et al*. 2005; Sukandar *et al*. 2016; Umaru *et al*. 2020; Wei *et al*. 2019; Yahia *et al*. 2020; Zahra & Iskandar 2017).

## MATERIALS AND METHODS

### Data Retrieval

This research was aimed to target the HPV16 E6 oncoprotein. The target protein’s amino acid sequence was obtained from UniProt (https://www.uniprot.org/uniprot) with ID P03126. The 3D structure of protein was modelled using I-TASSER webserver (https://zhanglab.dcmb.med.umich.edu/I-TASSER/). The 3D model was chosen based on the rank of the generated model, with the highest C-score value and TM-score. The compound data comes from various plant types commonly consumed in Indonesia. There are 476 bioactive compounds derived from 18 plant sources known to have antiviral, antibacterial, antifungal and antimicrobial properties (Abdallah *et al*. 2016; Abdel-Moneim *et al*. 2013; Amalina *et al*. 2013; Anwar *et al*. 2009; Asika *et al*. 2016; Badgujar *et al*. 2014; Beyzi *et al*. 2017; Choi *et al*. 2016; Chung 2009; Dertyasasa & Tunjung 2017; Ekpenyong *et al*. 2015; El-Saber Batiha *et al*. 2020; Fagodia *et al*. 2017; Figueirinha *et al*. 2008; González-Molina *et al*. 2010; Kadam & Lele 2017; Laribi *et al*. 2015; Li *et al*. 2017; Mahmood *et al*. 2016; Majdi *et al*. 2020; Mohammed *et al*. 2016; Pandey *et al*. 2016; Patil *et al*. 2009; Patra *et al*. 2020; Piras *et al*. 2013; Rattanachaikunsopon & Phumkhachorn 2010; G. Shah *et al*. 2011; Srivastava *et al*. 2005; Sukandar *et al*. 2016; Umaru *et al*. 2020; Wei *et al*. 2019; Yahia *et al*. 2020; Zahra & Iskandar 2017). The structure of all these potential compounds was mined from PubChem (https://pubchem.ncbi.nlm.nih.gov/) in structural data file (SDF) format (Putra *et al*. 2023; Putra 2018). Two drug compounds are used as controls in this study: vorinostat (CID: 5311) and mitomycin C (CID: 5746), currently used in cervical cancer therapy. Those drugs were also downloaded for their 3D structure in the SDF format.

### Drug-likeness Screening

All gathered compounds underwent testing against the Lipinski rule of five parameters to evaluate their pharmacological characteristics as our previous study (Hidayatullah *et al*. 2021; Putra & Rifa’i 2020). The Lipinski test was conducted utilising the Supercomputing Facility for Bioinformatics and Computational Biology at IT Delhi server (http://www.scfbio-iitd.res.in/software/drugdesign/lipinski.jsp) (Jayaram *et al*. 2012). Subsequently, all potential compounds meeting Lipinski’s criteria will be subjected to minimisation and converted to the AutoDock format using the PyRx programme integrated with the OpenBabel GUI (Mirzaei *et al*. 2015).

### Molecular Docking Process

The docking procedure is conducted utilising AutoDock Vina, integrated with PyRx (https://pyrx.sourceforge.io/) (Trott & Olson 2009). Our docking protocol encompasses the entire structure of the target protein. The molecular coverage area is delineated by dimensions of 44.4289 × 38.7535 × 71.0904 Angstroms, with a central coordinate set at 63.4878 × 63.4683 × 56.2079. The primary docking outcomes include the compound’s affinity expressed in kcal/mol, the location of the binding site, and the subsequent visualisation of protein-ligand interactions (Widiastuti *et al*. 2023; Putra *et al*. 2021).

### Visualisation Process

The visualisation procedure comprises two distinct stages: initially, a 3D visualisation is employed to gain a comprehensive understanding of potential compound binding sites on the E6 protein. Subsequently, a 2D visualisation is employed to discern interactions within each protein-ligand complex (Hidayatullah *et al*. 2020; Putra *et al*. 2019). The 3D visualisation step is executed using PyMOL (https://pymol.org/2/), while the 2D visualisation step is conducted using LigPlot+ 2.1 (https://www.ebi.ac.uk/thornton-srv/software/LigPlus/).

### Molecular Dynamics Simulation

Ligands exhibiting the lowest binding affinity scores were chosen for molecular dynamics (MD) simulation against the E6 protein. Simulation parameters were configured to mirror normal physiological conditions, including a temperature of 37°C, pressure of 1 atm, pH of 7.4 and a salt content of 0.9%. The MD simulation was conducted for a duration of 1,000 picoseconds. The simulation process was executed using the md_run macro programme, followed by subsequent analysis using md_analyze and md_analyeres (Hidayatullah *et al*. 2023).

## RESULTS

**Figure 1:**
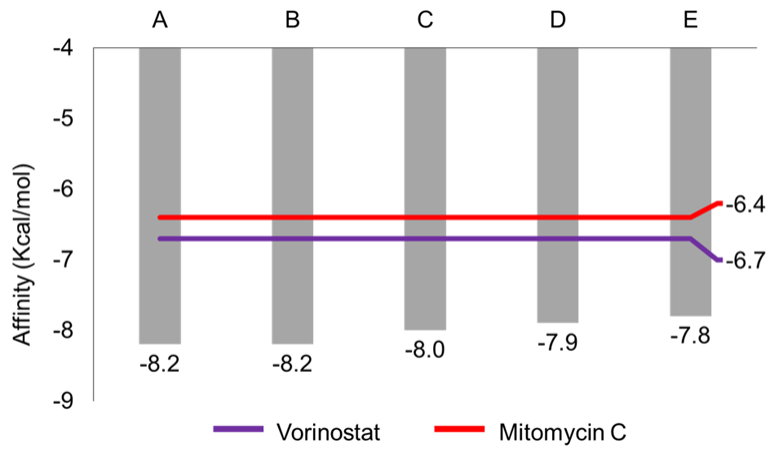
The affinity values of the top five compounds and controls, as determined by the docking results: (A) asarinin, (B) thiazolo[3,2-a]benzimidazol-3(2H)-one, 2-(2-fluorobenzylideno)-7,8-dimethyl, (C) ellagic acid, (D) magnoflorine, and (E) galbacin.

The molecular docking results revealed that the top five compounds tested exhibited affinity values surpassing those of the two drug controls (mitomycin C = −6.4 kcal/mol, vorinostat = −6.7 kcal/mol). Specifically, the top five compounds identified are asarinin, thiazolo[3,2-a]benzimidazol-3(2H)-one,2-(2-fluorobenzylideno)-7,8-dimethyl, ellagic acid, magnoflorine and galbacin. These compounds demonstrated affinities ranging from −7.8 kcal/mol to −8.2 kcal/mol, approximately 19% to 25% lower than both controls (Fig. 1).

**Figure 2:**
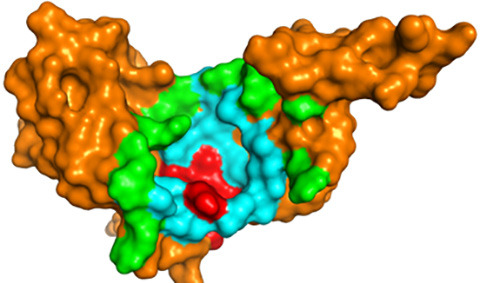
The 3D structure of target protein, HPV16 E5 protein. Red indicates the main helix structure of the E6 protein and cyan indicates the E6AP/IRF3 binding pocket, green indicates the E6 pocket residue.

**Figure 3:**
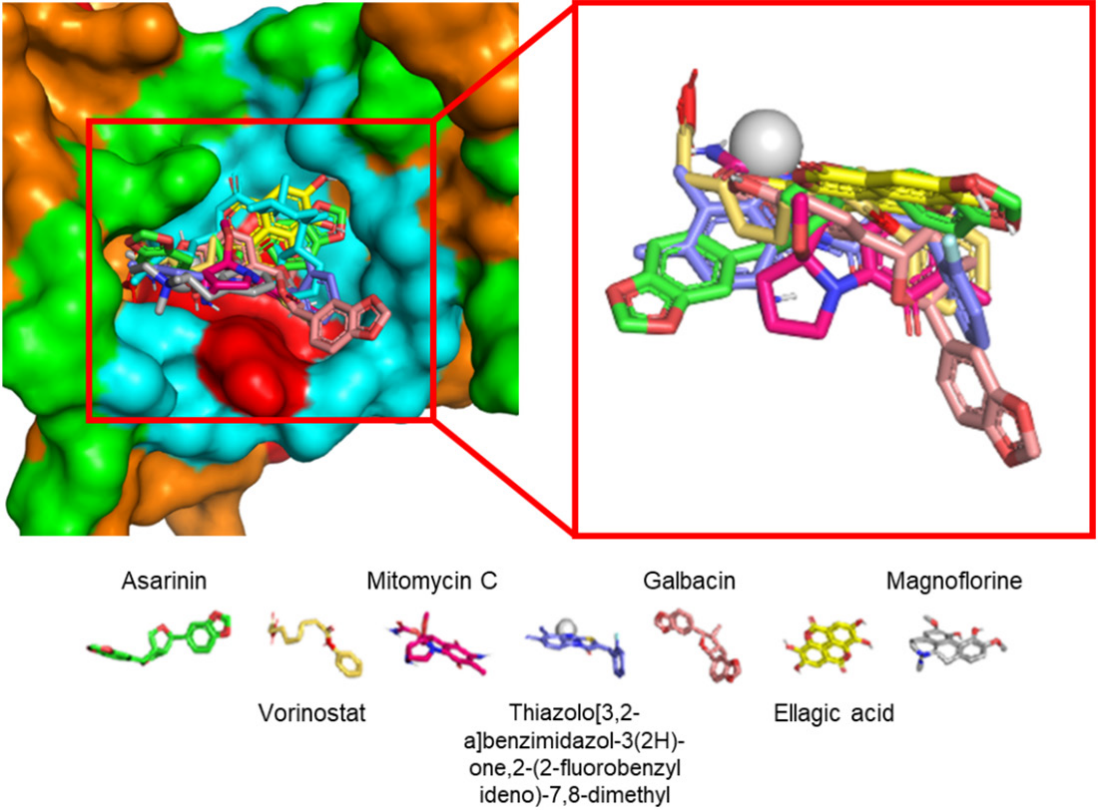
All tested compounds occupied the same binding site. Red indicates the main helix structure of the E6 protein, cyan indicates the E6AP/IRF3 binding pocket and green indicates the E6 pocket residue.

Both 2D and 3D visualisation outcomes depict that all top five compounds and controls were localised within a singular binding site. This binding site is situated adjacent to the main helix of the target protein, believed to be the binding site of the LxxLL motif of E6AP and IRF3 (Fig. 2). The dominant interaction formed in protein-ligand complexes is hydrophobic contact, with 86.5% of all interactions formed. Hydrogen bonds are only found in two potential compounds: ellagic acid, magnoflorine and drug controls. The hydrogen bonds formed by ellagic acid and magnoflorine have donor-acceptor distances ranging from 2.67Å–3.03Å and 2.94Å. The hydrogen bonds formed on vorinostat and mitomycin C have donor-acceptor distances ranging from 2.77Å–3.10Å and 3.13Å–3.16Å, respectively (Table 1; Fig. 3).

Based on the identification results, the E6 protein from various sources exhibits three main residue groups associated with E6AP binding: the E6AP binding pocket, E6 pocket and primary alpha helix residue (Table 2). Interestingly, there are interconnections among these residue groups, suggesting their involvement in forming the E6AP binding site. Specifically, the residues within the E6 pocket are found to be part of the E6AP binding pocket. Additionally, Leu74 and Ser78, classified as primary alpha helix residues, are identified to interact directly with E6AP. Notably, Tyr77 is a residue that is part of all three groups simultaneously. Furthermore, the identification of IRF3’s LxxLL binding site reveals that this motif’s binding site aligns perfectly with the E6AP binding pocket (Table 2).

**Figure 4:**
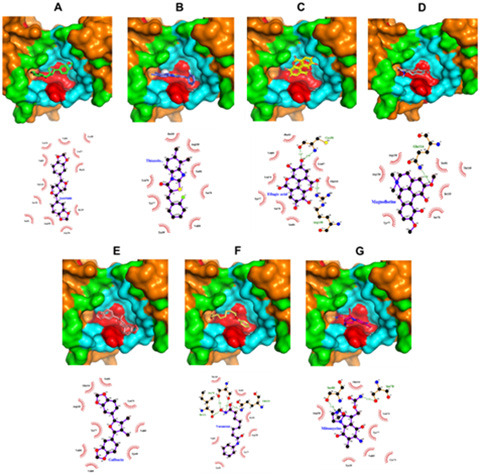
The 3D and 2D structure visualisation of binding HPV16 E6 protein and top five compounds molecule: (A) asarinin, (B) thiazolo[3,2-a]benzimidazol-3(2H)-one, 2-(2-fluorobenzylideno)-7,8-dimethyl, (C) ellagic acid, (D) magnoflorine, (E) galbacin, (F) vorinostat and (G) mitomycin C.

The results of 2D visualisation (Table 1; Fig. 4) highlight several conserved residues, including Arg138, Ser81, Gln114, Ser78 and Tyr77. Specifically, Ser81 and Gln114 are identified as binding site residues of E6AP (cyan), while Ser78 and Tyr77 are residues of the main helix structure (red), and Arg138 belongs to the E6 pocket (green). Interestingly, these residues exhibit overlap among the three groups; for instance, Arg138, besides being part of the E6 pocket, also contributes to the E6AP binding pocket, while Tyr77 is involved in both the E6 pocket and E6AP binding pocket, as well as the main helix structure. Additionally, the 2D visualisation results indicate that 89% of the residues interacting with the target protein are E6AP binding pocket residues. Notably, asarinin, the compound with the highest affinity, interacts most extensively with target protein residues compared to other potential compounds and drug controls (involving 12 residues). Overall, the analysis suggests that the residues associated with potential compounds and drug controls share more than 80% identity.

Asarinin primarily interacts with the E6AP binding pocket via hydrophobic contacts facilitated by its primary structure. Some key residues involved in the interaction with asarinin include Cys58, Leu57, Arg138, Ser81, Ser78, Gln114, Val69, Leu74 and Val60. It’s worth noting that while Arg138 is part of the E6 pocket, Ser78 and Leu74 are also components of the E6 main helix. Additionally, the binding sites of asarinin and both controls exhibit overlap, sharing common residues such as Arg138, Ser81, Ser78, Gln114, Val69 and Leu74.

Thiazolo[3,2-a]benzimidazol-3(2H)-one,2-(2-fluorobenzylideno)-7,8-dimethyl also extensively interacts with the E6AP binding pocket primarily through its primary structure and a hydroxyl group at C7, facilitated by hydrophobic contacts. Critical residues involved in this interaction include Ile135, Arg138, Ser81, Ser78, Tyr39, Tyr77 and Leu75. While Arg138 is part of the E6 pocket, Ser78 and Leu74 belong to the main helix, and Tyr77 is involved in the E6AP/IRF3 binding pocket, E6 pocket, and the main helix simultaneously. These residues are shared with both drug controls, suggesting a potentially significant role in the compound’s mechanism of action.

Ellagic acid, one of the potential compounds, forms hydrogen bonds with target protein residues. It interacts predominantly with the E6AP/IRF3 binding pocket, overlapping with some E6 pocket and primary helix residues, primarily through its main structure and hydroxyl groups on C11, C5, C7, C13, C12, C6, C8 and C14, facilitated mainly by hydrophobic contacts. Additionally, the hydroxyl groups on C13 and C12 establish moderate to low hydrogen bonds with Cys58 and Arg138, respectively. Critical residues involved in this interaction include Leu57, Gln114, Ser81, Ser78, Tyr77, Leu74, Val69, Cys58 and Arg138, which are commonly shared with the binding sites of vorinostat and mitomycin C.

Similarly, magnoflorine interacts with the target protein through hydrophobic contacts and hydrogen bonds. It exhibits nearly identical interactions with the previously mentioned compounds, primarily interacting with the E6AP/IRF3 binding pocket and some overlapping E6 pocket and primary helix residues. Magnoflorine’s interactions involve hydrophobic contacts with its main ring structure, methoxy groups on C14 and C17, and a hydroxyl group on C13. The methoxy group on C14 additionally forms a moderate hydrogen bond (2.94Å) with Gln114. Critical residues in this interaction include Ser81, Ile135, Ser78, Tyr77, Arg136, Arg138 and Gln114, with all except Arg136 being identical to the binding site of the drug controls.

The last potent compounds in this study, galbacin, predominantly interact with the E6AP/IRF3 binding pocket, along with some overlapping E6 pocket and main helix residues, primarily through hydrophobic contacts, which is consistent with the behavior observed in previous compounds. Notable critical residues involved in this interaction include Leu74, Val69, Tyr39, Val60, Val38, Tyr77, Arg138, Gln114 and Ser81, all of which are identical to the binding site of the drug controls, except for Val60 and Val38.

Regarding the control compounds used in this study, vorinostat and mitomycin C, their interactions with the binding site closely mirror those of the potential compounds. Specifically, vorinostat’s binding site residues exhibit a similarity of approximately 77%, with the residues interacting with the rest of the potential compounds from various natural sources. Similarly, mitomycin C’s binding site interactions are nearly 88% identical to those of the top five compounds. Both controls share about 67% of their binding site residues, including some highly conserved residues such as Arg138, Tyr77, Ser81 and Gln114.

Based on the docking and visualisation results, we selected the top two compounds and vorinostat as a control for further analysis using molecular dynamics. Essential parameters such as potential energy, root mean square deviation (RMSD) and root mean square fluctuation (RMSF) values were monitored over 1,000 picoseconds of simulation to provide insights into the dynamic behaviour of the protein-ligand complexes.

**Figure 5:**
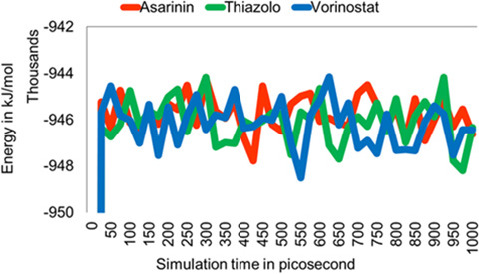
Total potential energy of the system among HPV16 E6 and ligands interaction over a 1,000 picoseconds simulation.

The total potential energy profiles of three molecules during a molecular dynamics simulation (Fig. 5) reach a relatively stable equilibrium after initial fluctuations. The graph provides insights into the relative stability and dynamic behavior of these molecules within the simulated environment. The molecules continue to exhibit minor energy fluctuations, reflecting their ongoing movements and interactions at the atomic level. However, the overall energy remains within a defined range, suggesting that the molecules have found stable configurations or interactions within the simulated environment.

**Figure 6:**
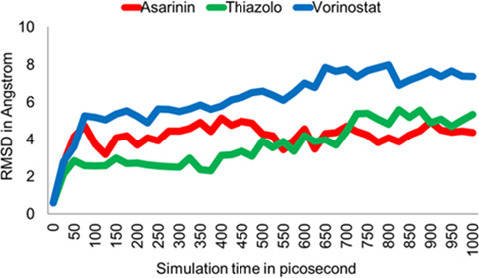
RMSD plot for HPV16 E6 and Asarinin (red line), Thiazolo (green line), and Vorinostat (blue line) complexes over a 1,000 picoseconds of simulation.

The RMSD was computed utilising 1,000 picoseconds of simulation to evaluate the flexibility and overall stability of the docked complexes (Fig. 6). The value for E6-asarinin was 4.163Å ± 0.758Å, with a minimum of 0.600Å and a maximum of 5.151Å. The E6-thiazolo[3,2-a]benzoidazole-3(2H)-one,2-(2fluorobenzylideno)-7,8-dimethyl complex has a mean value of 3.641Å ± 1.203Å, ranging from 0.600Å to 5.594Å. The mean value of E6-vorinostat was 6.206Å ± 1.484Å, with a minimum value of 0.591Å and a maximum value of 7.999Å.

The RMSD plot illustrates that both candidate compounds exhibit significantly lower RMSD values than controls. In the case of the asarinin complex, after initial oscillations between 0 ps and 150 ps, it stabilises around 4. On the other hand, the thiazolo[3,2-a]benzoidazole-3(2H)-one,2-(2-fluorobenzylideno)-7,8-dimethyl complex demonstrates stability between 50 ps and 400 ps, maintaining an RMSD around 3 before showing a slight increase in values towards the end of the simulation, ranging from 5 to 5.5. In contrast, the vorinostat complex continuously increases RMSD values throughout the simulation period.

Based on the trajectory graph, the E6-asarinin complex appears to be the most stable protein-ligand combination, followed by the thiazolo[3,2-a] benzoidazole-3(2H)-one,2-(2-fluorobenzylideno)-7,8-dimethyl complex, which remains stable up to 400 ps before displaying a slight increase in RMSD values. Meanwhile, the vorinostat complex exhibits a steady rise in RMSD values throughout the simulation, indicating potential instability in the protein-ligand interaction over time.

**Figure 7:**
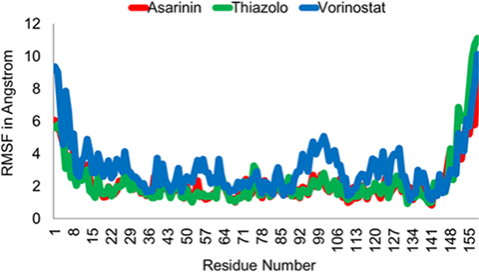
RMSF plot for HPV16 E6 and asarinin (red line), thiazolo (green line), and vorinostat (blue line) complexes residues over a 1,000 picoseconds simulation.

The RMSF plot (Fig. 7) highlights that, aside from the N- and C-termini, minimal variations were observed across the residues for both potential compounds throughout the simulation period. This indicates a well-organised synthesis of these two protein-ligand complexes. In contrast, the vorinostat complex consistently exhibited higher RMSF values and more significant oscillations than the other two compounds investigated. This suggests that vorinostat was the least stable among the three substances during the molecular dynamics simulation. However, it’s worth noting that all compounds displayed low RMSF values for the residues with which they interacted, indicating relatively stable interactions despite differences in overall stability.

## DISCUSSION

One of the crucial roles of E6 protein in cervical cancer development is the degradation of p53, which stimulates cell proliferation and prevents apoptosis, also the degradation of DLG1 or NFX1 hosts, which will lead to upregulation of human telomerase reverse transcriptase (hTERT) and ends in cell immortalisation (Bernard *et al*. 2011; Doorbar *et al*. 2012; Sekaric *et al*. 2008; Van Doorslaer & Burk 2010). However, to carry out these functions, the E6 protein cannot stand alone but forms a complex with E6AP (Martinez-Zapien *et al*. 2016; Sailer *et al*. 2018). E6, E6AP and p53 complexes already visualised by PDB entry ID 4XR8 (Martinez-Zapien *et al*. 2016). The E6–E6AP complex is connected through a helix linker structure known as leucine (L)-rich motifs (LxxLL), which binds to residues around the E6 protein main alpha helix, the main target in this study. The disruption between E6 protein and E6AP is predicted to inhibit the E6-E6AP complex formation, lowering the degradation rate of p53. In this experiment, the disturbance was carried out with potent natural compounds expected to occupy the same binding site as E6AP to act as a competitive inhibitor. A similar interaction mechanism also occurs in the formation of the E6-IRF3 complex. Interestingly, the E6-IRF3 complex is connected by the interaction between IRF3’s LxxLL motif and the hydrophobic pocket E6 protein around its main alpha helix (Reiser *et al*. 2011; Ronco *et al*. 1998; M. Shah *et al*. 2013). Thus, the E6-E6AP complex formation disturbance by some potential compounds were expecting acting as a competitive inhibitor at the E6AP binding site. Furthermore, we hypothesised that the interference also occurs in the E6-IRF3 complex due to the LxxLL motifs formation, since the E6AP and IRF3 have identical binding sites.

The docking results showed that all of the top five compounds had lower affinity values than the two-drug controls, indicating that the interaction between the top five compounds with the target protein is more stable than the interaction between the controls and the target protein (Pantsar & Poso 2018; Trott & Olson 2009), and have a higher tendency to form interactions between top five compounds and target protein than drug controls (Ajay & Murcko 1995). The asarinin found in *Zanthoxylum* spp. and thiazolo[3,2-a]benzimidazol-3(2H)-one,2-(2-fluorobenzylideno)-7,8-dimethyl found in *Myristica fragrans* are two potential compounds that have the best affinity values based on docking result. Their lowest affinity is 8.2 kcal/mol, roughly 22.3% and 28.0% lower than vorinostat and mitomycin C, respectively. Thus, these tend to form a more stable interaction with the E6AP/IRF3 binding pocket on the E6 protein than the other natural compounds and drug controls. However, asarinin interacts with more E6AP/IRF3 binding pocket residues than thiazolo[3,2-a]benzimidazole-3(2H)-one,2-(2-fluorobenzylideno)-7,8-dimethyl (10 to 8 residues), Although the difference in the number of interactions is not significant, it is suspected that there is an effect on the stability of the interactions formed between each compound and the E6AP/IRF3 binding pocket. All interactions established between the two compounds and the E6AP/IRF3 binding pocket are characterised as hydrophobic interactions, which are inherently stronger at a molecular level compared to other intermolecular interactions such as hydrogen bonds or Van der Waals bonds (Atkins & de Paula 2010; Jeffrey 1997).

The two top-five compounds that form simultaneous hydrophobic interactions and hydrogen bonds are ellagic acid found in *Syzygium aromaticum* and magnoflorine found in *Nigella sativa*. The two compounds also had better affinity values than the controls. The docking results show that the hydrogen bonds formed in the two compounds are moderate, primarily electrostatic, and act as supporting bonds because they have a donor-acceptor distance in the 2.5Å–3.2Å range. As a whole, these compounds do not have better affinity than asarinin, which entirely relies on hydrophobic contacts (Jeffrey 1997).

The top five compounds and the two drug controls have binding sites on the E6 protein almost identical to each other, characterised by several conserved residues mentioned above; only three residues (4.7%) that exclusively interact with one compound, including Cys73, Ile111 and Thr140. The visualisation results in Fig. 2 confirmed the 2D visualisation results even further. The stable interaction between the top five compounds at the E6AP’s LxxLL motif binding site is predicted to inhibit the formation of the E6-E6AP complex that is crucial in the p53 degradation process by preventing the E6AP’s LxxLL domain from binding to its specific binding site. The interaction between E6AP’s LxxLL motif and E6 protein will trigger a conformational change of the E6 protein into a suitable and stable conformation to bind with p53, characterised by the formation of the p53 binding cleft (Sailer *et al*. 2018; Vande Pol & Klingelhutz 2013), inhibiting the degradation process of p53 because E6 and E6AP are not stable enough to be carried out the p53 binding and degradation process itself. It is predicted that the mechanism of action of p53 can still manageably running, mainly causing cells infection to apoptosis (Huibregtse *et al*. 1993; Martinez-Zapien *et al*. 2016).

In addition, the top five compounds’ interaction is also thought to affect the formation of the E6-IRF3 complex because the IRF3’s LxxLL motifs have a binding site that is identical to the LxxLL motif possessed by E6AP (M. Shah *et al*. 2013). The stable form of the E6-IRF3 complex is found exclusively in the HPV16 variant. The binding of the IRF3’s LxxLL motif to the E6 protein does not cause IRF3 degradation; instead, it suppresses the transactivation process, which will inhibit IFN-β transcription (Westrich *et al*. 2017). IRF3 has two LxxLL motifs in its N-terminal (140-LDELLG-145 (IRF3-LR1) and 192-LKRLLV-197 (IRF3-LR2)), and autoinhibitory domain (AD) that flank the IRF association domain (IAD) in its C-terminal (Chen & Royer 2010; R. Lin *et al*. 1999). When the E6 protein binds to IRF3 through its LxxLL motif, the E6 protein will change its conformation to interact with Ser-patches on IRF3. The interaction of E6 protein with Ser-patches on IRF3 will inhibit the activation process of IRF3 through a phosphorylation mechanism so that there is no co-activation process of IFN-β transcription with p300/CBP (Howie *et al*. 2009; M. Shah *et al*. 2013). In addition, the interaction between E6 and IRF3 will result in the phosphorylation of the AD domain by virus-induced kinase, thus keeping IRF3 in an inactivated state (Tummers & van der Burg 2015). Therefore, the disruption of E6-IRF3 complex formation by some potential compounds is thought to have an undisturbed signalling mechanism and activation of the IRF3 pathway so that the overall innate immune response performs better against HPV16 infection.

To deepen the analysis, we performing molecular dynamic simulation as our previous study (Hidayatullah *et al*. 2022). Asarinin and thiazolo[3,2-a] benzoidazole-3(2H)-one,2-(2-fluorobenzylideno)-7,8-dimethyl, the two of the top compounds have relatively lower RMSD and RMSF values than the control, indicating that the complex formed by the two compounds is more stable than vorinostat used as a control. The RMSF plot shows consistently low values for residues suspected to interact with these two compounds and control based on the results of 2D visualisation, indicating that these residues are critical in the active site of the HPV16 E6 protein’s hydrophobic groove and indicate stability in the residue region bound to the tested ligands (Aier *et al*. 2016; Zhao *et al*. 2015).

## CONCLUSION

The majority of cervical cancer cases are attributed to HPV16 infection, with the E6 protein serving as a key oncoprotein implicated in cancer development. Docking and visualisation results reveal that all of the top five compounds are concentrated within a single binding site on the E6 main hydrophobic groove, which coincides with the binding site of the E6AP and IRF3’s LxxLL motifs. These compounds are predicted to function as competitive inhibitors, potentially obstructing the formation of the E6-E6AP and E6-IRF3 complexes. By doing so, they may hinder the degradation of p53, consequently impeding cell proliferation and sustaining the innate immune response against HPV16 infection. Notably, asarinin and thiazolo[3,2-a]benzimidazole-3(2H)-one,2-(2-fluorobenzylideno)-7,8-dimethyl emerged as the most potent compounds, exhibiting the highest affinity value (−8.2 kcal/mol) and forming stable interactions with the E6 main hydrophobic groove. However, further investigations utilising in vivo or in vitro methods are warranted to validate the computational predictions presented in this study.

## Figures and Tables

**Table 1:** Docking and 2D visualisation result of top five compounds against HPV16 E6 protein.

Compounds	ΔG	Amino acid residue	Interactions (Å)
Asarinin (CID: 11869417)	−8.2 (kcal/mol)	Cys58; Leu57; Phe52; Ile135; Arg136; Arg138; Ser81; Ser78; Gln114; Val69; Leu74; Val60	Hydrophobic contact
*Zanthoxylum* spp. (bark)			

Thiazolo[3,2-a] benzimidazol-3(2H)-one,2-(2-fluorobenzylideno)-7,8-Tyr77; Leu74 dimethyl (CID: 1823738)	−8.2 (kcal/mol)	Ile135; Arg138; Ser81; Ser78; Val38; Tyr39;	Hydrophobic contact
*Myristica fragrans* (seeds)			

Ellagic acid (CID: 5281855)	−8.0 (kcal/mol)	Leu57; Gln114; Ser81; Ser78; Tyr77; Leu74; Val69; Phe52	Hydrophobic contact
*Syzygium aromaticum* (flowers)		Cys58	Hydrophobic contact; Hydrogen bond (2.67); Hydrogen bond (2.99)
		Arg138	Hydrophobic contact; Hydrogen bond (3.03)

Magnoflorine (CID: 73337)	−7.9 (kcal/mol)	Ser81; Ile135; Ser78; Tyr77; Arg136; Arg138	Hydrophobic contact
*Nigella sativa* (seeds)		Gln114	Hydrophobic contact; Hydrogen bond (2.94)

Galbacin (CID: 234441)	−7.8 (kcal/mol)	Leu74; Val69; Tyr39; Val60; Val38; Tyr77; Arg138; Gln114; Ser81	Hydrophobic contact
*Myristica fragrans* (seeds)			

Vorinostat (CID: 5311)	−6.7 (kcal/mol)	Ile135; Arg138; Tyr77; Val69; Thr140; Ser81	Hydrophobic contact
DHAC inhibitor (Drug)		Gln114	Hydrophobic contact; Hydrogen bond (3.10)
		Ile111	Hydrophobic contact; Hydrogen bond (2.77)
		Ser78	Hydrophobic contact; Hydrogen bond (2.94); Hydrogen bond (2.95)

Mitomycin C (CID: 5746)	−6.4 (kcal/mol)	Leu74; Tyr77; Cys73; Val69; Tyr39; Arg138; Gln114	Hydrophobic contact
Anti-cancer drug		Ser78	Hydrophobic contact; Hydrogen bond (3.13)
		Ser81	Hydrophobic contact; Hydrogen bond (3.16)

**Table 2:** Identification result of critical binding site and residues of HPV16 E6 protein.

Residues group	E6 residues
E6AP binding pocket	Arg17, Lys18, Val38, Tyr39, Asp56, Leu57, Val60, Arg62, Val69, Leu74, Tyr77, Ser78, Ile80, Ser81, Arg84, His85, Ser87, Tyr88, Gln98, Gln99, Leu107, Arg109, Glu114, Asn134, Ile135, Arg136, Gly137, Arg138 (M. Shah *et al*. 2013)
E6 pocket	Arg62, Arg17, Arg109, Arg136, Arg138, Arg84, Tyr77 (Ricci-López *et al*. 2019)
Main alpha helix	Lys72, Cys73, Leu74, Lys75, Phe76, Tyr77, Ser78, Lys79, Ile80, Ser81, Glu82, Tyr83, Arg84, His85 (Rietz *et al*. 2016; Vande Pol & Klingelhutz 2013)
IRF3 binding pocket	Lys18, Val38, Tyr39, Leu57, Val60, Arg62, Val69, Leu74, Tyr77, Ser78, Ile80, Ser81, Arg84, His85, Arg109, Glu114, Arg138 (M. Shah *et al*. 2013)
